# Facilitators and Barriers to the Adoption of Telemedicine During the First Year of COVID-19: Systematic Review

**DOI:** 10.2196/31752

**Published:** 2022-01-04

**Authors:** Clemens Kruse, Katharine Heinemann

**Affiliations:** 1 School of Health Administration Texas State University San Marcos, TX United States

**Keywords:** telemedicine, pandemic, technology acceptance, COVID-19, digital health, telehealth, health policy, health care

## Abstract

**Background:**

The virulent and unpredictable nature of COVID-19 combined with a change in reimbursement mechanisms both forced and enabled the rapid adoption of telemedicine around the world. Thus, it is important to now assess the effects of this rapid adoption and to determine whether the barriers to such adoption are the same today as they were under prepandemic conditions.

**Objective:**

The objective of this systematic literature review was to examine the research literature published during the COVID-19 pandemic to identify facilitators, barriers, and associated medical outcomes as a result of adopting telemedicine, and to determine if changes have occurred in the industry during this time.

**Methods:**

The systematic review was performed in accordance with the Kruse protocol and the results are reported in accordance with the PRISMA (Preferred Reporting Items for Systematic Reviews and Meta-Analyses) guidelines. We analyzed 46 research articles from five continents published during the first year of the COVID-19 pandemic that were retrieved from searches in four research databases: PubMed (MEDLINE), CINAHL, Science Direct, and Web of Science.

**Results:**

Reviewers identified 25 facilitator themes and observations, 12 barrier themes and observations, and 14 results (compared to a control group) themes and observations. Overall, 22% of the articles analyzed reported strong satisfaction or satisfaction (zero reported a decline in satisfaction), 27% reported an improvement in administrative or efficiency results (as compared with a control group), 14% reported no statistically significant difference from the control group, and 40% and 10% reported an improvement or no statistically significant difference in medical outcomes using the telemedicine modality over the control group, respectively.

**Conclusions:**

The pandemic encouraged rapid adoption of telemedicine, which also encouraged practices to adopt the modality regardless of the challenges identified in previous research. Several barriers remain for health policymakers to address; however, health care administrators can feel confident in the modality as the evidence largely shows that it is safe, effective, and widely accepted.

## Introduction

### Rationale

The virulent nature of COVID-19 forced social distancing and a decrease of in-person visits to clinics around the world. Telemedicine presented health care providers with solutions that enabled a social-distancing window into the clinical environment and a continuation of the doctor-patient relationship.

Telemedicine is defined by the World Health Organization as healing from a distance through information communications technologies by all health care professionals for the “exchange of valid information for diagnosis, treatment and prevention of disease and injuries, research and evaluation” [1]. Telemedicine is not a perfect means of patient care; however, it offers great advantages to overcome geographical barriers to improve health outcomes [1]. Validated and peer-reviewed international statistics are elusive on adoption figures, but a recent question-and-answer session indicates overall low adoption of telemedicine internationally [2]. In the United States, prior to the pandemic, telemedicine had only been adopted by 8% of providers [3]. Providers have recognized wide acceptance of telemedicine by patients; however, prior to the desperate circumstances of COVID-19, they had not been willing to adopt telemedicine on a wide scale [4]. The largest challenges to the adoption of telemedicine were identified as technically challenged staff, resistance to change, cost, reimbursement, and education level of the patient [5]. Telemedicine saves patients time, consultation fees, and travel expenses [6]. However, telemedicine requires users at both ends to possess certain levels of technological skills such as those required to enable video teleconferencing [7]. Fortunately, some countries enacted legislation to expand the adoption of telemedicine. For example, in the United States, telemedicine was not easily reimbursed by federal programs until the Coronavirus Aid, Relief, and Economic Security (CARES) Act legislation [8], which greatly increased reimbursement mechanisms for the telemedicine modality. This change in reimbursement structure should not be ignored, and it most likely provided a significant catalyst to the adoption of telemedicine.

A large number of articles were published in the first 12 months of the pandemic (February 2020 to February 2021) on the rapid implementation efforts of telemedicine to enable clinics and hospitals to continue to see patients and care for their needs [9,10]. However, providers acknowledge some of the shortfalls inherent to this modality, such as lack of technical infrastructure, cost, lack of technical staff, computer literacy of both staff and patients, and a negative impact on the patient-to-provider relationship [4,11-13]. A systematic review performed in 2020 on telemedicine and COVID-19 evaluated 44 articles along four service lines and identified 10 themes of efficiency [14]. However, the authors did not evaluate facilitators and barriers to adoption or health outcomes. Another systematic review [5] was performed in 2016 on the barriers to the adoption of telemedicine worldwide, which evaluated 30 articles across all service lines in all countries; however, it also did not evaluate facilitators or health outcomes.

Although analyses have been published that highlight the advantages to the adoption of telemedicine, with an 8% adoption rate in the United States, the conclusions of these previous studies may not be as robust as possible. The circumstances presented by the pandemic have encouraged wider adoption of this modality of care. Therefore, with proper systematic review techniques, reviewer observations this far into the pandemic will undoubtedly be more robust and widely applicable to medicine.

### Objectives

The purpose of this systematic review was to evaluate the facilitators and barriers to the adoption of telemedicine worldwide, including an analysis of health outcomes and patient satisfaction. A brief comparison of the results of this review with those of reviews performed prior to COVID-19 was further performed to identify changes in these factors in light of the pandemic. 

## Methods

### Protocol and Registration

The Kruse protocol for writing a systematic review was followed, and the findings are reported in accordance with the PRISMA (Preferred Reporting Items for Systematic Reviews and Meta-Analysis) guidelines [15,16]. This systematic review was registered in PROSPERO on August 2, 2021 (ID CRD42021235933).

### Eligibility Criteria

The search parameters were established to find articles published in 2020 and 2021 concerning telemedicine in all aspects of care and for all ages of patients, published in peer-reviewed journals, using any method of study (mixed method, quantitative, and qualitative). Other systematic reviews were excluded because we wanted to compare our results to these previous reviews without confounding the findings. The Johns Hopkins Nursing Evidence-Based Practice Rating Scale (JHNEBP) was used to assess the quality of all articles analyzed [17]. Any studies below level IV C were discarded due to poor quality.

### Information Sources

Four research databases were searched: PubMed (MEDLINE), CINAHL (excluding MEDLINE), Web of Science, and Science Direct. We also performed a journal-specific search of the Journal of Medical Internet Research.

### Search Strategy

Google Scholar was used to determine the general trends of publication on this topic previously and to collect key terms from published articles. These key terms were entered into the US Library of Medicine’s Medical Subject Headings (MeSH) to create an exhaustive search string using Boolean terms. The actual search string used was: (telemedicine OR telehealth OR “mobile health” OR mhealth OR ehealth) AND (COVID-19 OR coronavirus). The same search string was used in all databases. Similar filters were used in each database (not all filters are the same between databases).

### Study Selection Process

Once the search string was entered into each database, we filtered the results and screened abstracts for applicability. Although filters for the four research databases differ, we generally filtered for the date range (2020-2021), scholarly journals (no theses or opinions), and “full text” to ensure that we would have access to the entire article. Articles were rejected for a variety of reasons: protocol (no results to analyze); opinion (no data); reviews; did not use telemedicine; or did not contribute to our objective statement of identifying facilitators, barriers, or effects on patient satisfaction. The κ statistic was calculated to identify the level of agreement between reviewers [18].

### Data Collection Process

An Excel spreadsheet was used as a data-extraction tool to collect data for reporting and analysis. This spreadsheet was standardized according to the Kruse protocol [15]. We held three consensus meetings to screen abstracts, analyze articles, and discuss possible themes. After the second consensus meeting, we performed a narrative analysis to identify themes in the articles analyzed [19]. Because there were only two authors on this project, both authors analyzed all articles (n=46).

### Data Items

In accordance with the Kruse protocol, PRISMA standard, and JHNEBP, the following fields were collected: database source; date of publication; journal; authors; study title; PICOS (participants, intervention, results, outcomes, study design); sample size; bias within study; effect size; country of origin; statistics used; quality metrics from the JHNEBP scale; and reviewer observations as they relate specifically to the objective statement in areas of patient satisfaction, and facilitators and barriers to adoption [15,17,20]. All data items were independently collected and discussed in subsequent consensus meetings.

### Risk of Bias Within and Across Studies

The JHNEBP rating scale was used for assessment of bias within and across studies. Observations of bias and methodological weaknesses were noted [17]. The JHNEBP ratings also provided insight into bias because poor-quality results can limit the external validity of the experiment.

### Summary Measures

Because we included mixed methods and qualitative studies, we were unable to standardize summary measures as would be performed in a meta-analysis.

### Additional Analyses

We performed a narrative, or thematic, analysis of the observations to convert them into themes (common threads between articles) [19]. We calculated the frequency of occurrence of both themes and individual observations and report these in a series of affinity matrices (tables). This technique was used to identify the statistical probability for identifying each theme, which does not identify a level of importance but rather identifies a frequency of mention of these themes in the literature during the period of observation.

## Results

### Study Selection

**Figure 1:**
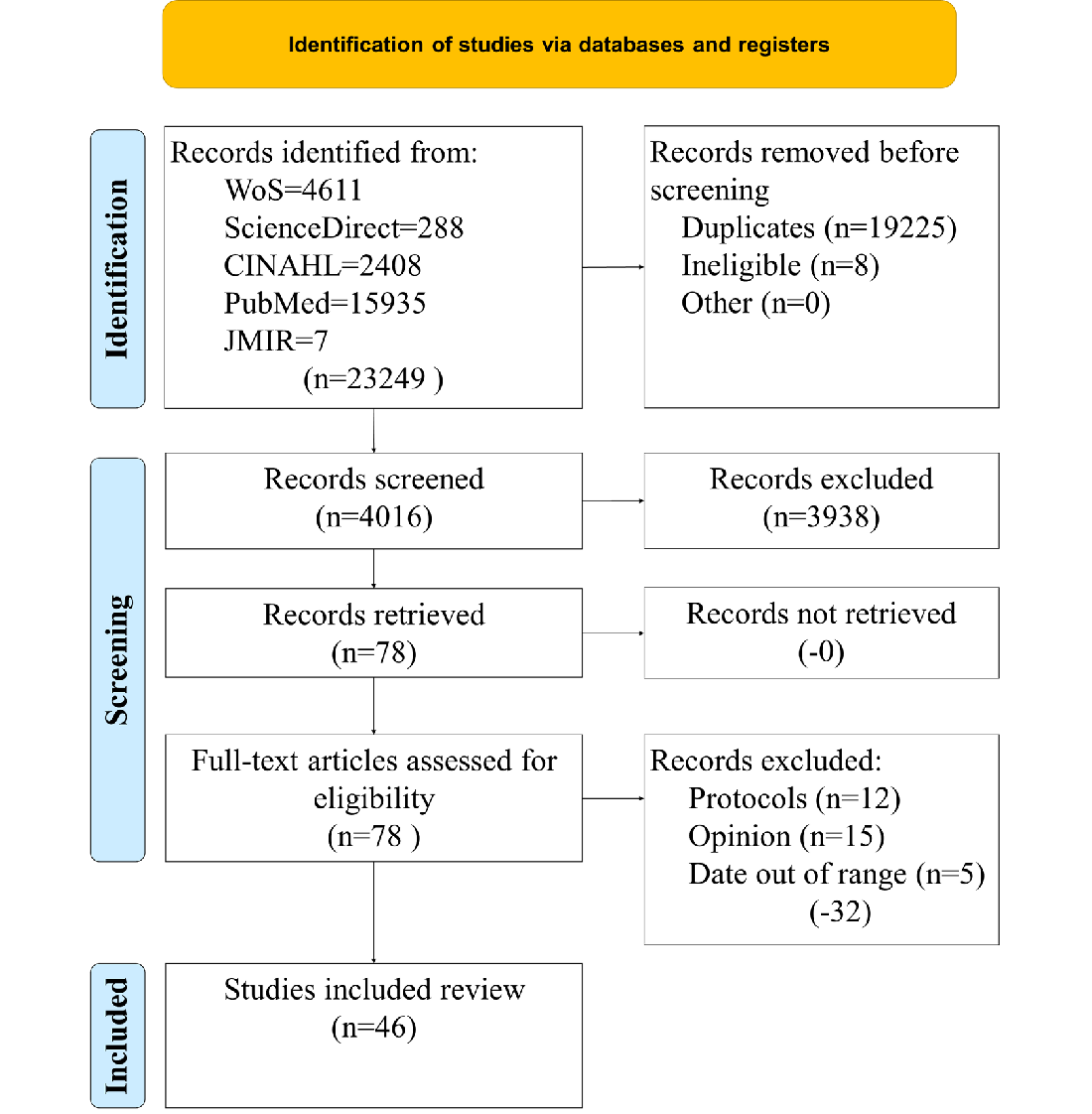
Article search and selection process. WoS: Web of Science.

The database search and study selection process are illustrated in Figure 1. The κ statistic was 0.95, indicating almost perfect agreement between reviewers [18,21]. Several studies made it through all filters, but were still eliminated because they were protocols (no results), opinions, out of the date range, or other systematic reviews.

### Study Characteristics

Reviewers collected study characteristics identified by the PRISMA standard such as PICOS (see Table 1). Of the 46 six studies analyzed over the 15-month period, 2 (4%) involved adolescents, 6 (13%) involved adults >60 years, and 38 (83%) involved adults >18 years as participants. Most participants were current or former patients who agreed to participate in studies. More than half the interventions were mobile health (mHealth), telephone/televideo, or eHealth (26/46, 56%). The rest were interventions involving telemonitoring, patient portals, telecoaching, web chat, and social media, which could be cross-platform. In these 46 studies, 18 resulted in a positive outcome over a control group (23%), 12 of which involved medically measured outcomes (21%) as opposed to clinical and administrative outcomes. Only 9 of the 46 (20%) studies resulted in no statistically significant difference between the intervention and control groups, which means that positive results could be obtained through telemedicine commensurate with those obtained using traditional means of care. Four articles analyzed were published in 2021 [22-25], with the remaining 42 articles published in 2020 [26-67]. Further explanation of the results and medical outcomes can be found the Additional Analysis subsection.

**Table 1:** Characteristics of the included studies according to the PICOS (Participants, Intervention, Results, Outcomes, and Study Design) structure.

Study	Participant	Intervention	Results (compared to the control group or other studies)	Medical outcomes	Design
Ben-Arye et al [22]	Adult patients (>18 years) undergoing adjuvant, neoadjuvant, or palliative treatment for solid tumors	eHealth	Improved compliance/adherence	Not reported	Prospective, controlled, and nonrandomized study
Yu et al [25]	Older adult patients (50% >60 years, 60% women, 68% one-time telehealth users) and 45 physicians	Telephone or televideo	Improved patient satisfaction	Not reported	Cross-sectional
Richards et al [24]	Adult respondents from a neurosurgical outpatient clinic (mean age 63 years, 50.3% men)	Telephone or televideo	Improved patient satisfaction	Not reported	Qualitative
Kurihara et al [23]	Adult patients with Parkinson disease (61% women, mean age 67 years) at Fukuoka University Hospital	Telemedicine self-testing	No control group (nonexperimental)	Not reported	Cross-sectional
Alkire et al [26]	Adults (Gen X, Millennial)	Patient portals	No control group (nonexperimental)	Not reported	Nonexperimental
Ballin et al [27]	Older adults, 70-year-old men, and women with central obesity	Supervised and web-based	No significant difference; decreased fat mass	Improved in at least one area: decreased fat mass	Randomized controlled trial
Banbury et al [28]	Adults >50 years with at least one chronic condition	Telemonitoring	Telemedicine improved results compared to control: companionship, emotional support, health literacy, self-management	Not reported	Mixed methods, quasiexperimental, nonrandomized trial
Barnett et al [29]	Adults (22-27 years; 10 men, 10 women), clients of an alcohol and drug counseling service across Australia, and 8 counselors	Webchat	No control group (nonexperimental)	Not reported	Qualitative study, nonexperimental
Batalik et al [30]	Adult cardiac rehabilitation patients	Home-based telerehab	No statistically significant difference	No statistically significant difference	Randomized controlled trial
Beller et al [31]	Adult patients scheduled for video visits through the University of Virginal urology departments	Televideo	No control group (nonexperimental)	Not reported	Cohort
Bernabe-Ortiz et al [32]	Adult participants from a randomized clinical trial on a 1-year mHealth^a^ intervention on blood pressure and body weight 4 years postcompletion	mHealth	Telemedicine improved results compared to control: decreased fat mass	Improved in at least one area; decreased body weight	Retrospective study of a randomized clinical trial
Bilgrami et al [33]	Adults with inflammatory bowel disease	Telemedicine self-testing	No statistically significant difference	No statistically significant difference	Randomized controlled trial
Broers et al [34]	Adult patients with cardiovascular disease	eHealth	No statistically significant difference; increased quality of life	Not reported	Randomized controlled trial
Cho et al [35]	Adult participants (30-59 years) with at least 2 conditions defined by the Third Report of the National Cholesterol Education Program expert panel (abdominal obesity, high blood pressure, high triglycerides, low high-density lipoprotein cholesterol, and high fasting glucose level)	mHealth	Telemedicine improved results compared to control (decreased fat mass)	Improved in at least one area: decreased fat mass, decreased body weight	Randomized controlled trial
Claes et al [36]	Adult patients with cardiovascular disease from 3 European hospitals	eHealth	Improved health behaviors	Not reported	Randomized controlled trial
Coorey et al [37]	Adults who had completed 12 months of follow-up from the Consumer Navigation of Electronic Cardiovascular Tools trial	eHealth	No control group (nonexperimental): improved self-management, improved health literacy	Not reported	Qualitative analysis of a randomized controlled study
Ding et al [38]	Adults (mean age 70.1 years) with chronic heart failure	Telemonitoring	Telemedicine improved results compared to controls: improved compliance/adherence	Not reported	Randomized controlled trial
Geramita et al [39]	Adult lung transplant recipients	mHealth	No statistically significant difference	Not reported	Randomized controlled follow-up study
Gong et al [40]	Adult hypertension	mHealth	Telemedicine improved results compared to controls: improved compliance/adherence	Improved in at least one area: reductions in blood pressure	Randomized controlled trial
Han et al [41]	Adults (<55 years) prepandemic (S1) and 273 follow-up surveys (S2); university-affiliated, and physicians	eHealth	No control group (nonexperimental): telemedicine improved results compared to controls, improved compliance/adherence	Not reported	Qualitative
Harding et al [42]	Adult caregivers with 837 patient assessment outcomes	mHealth	No control group (nonexperimental)	Not reported	Qualitative (pilot study)
Hsia et al [43]	Pediatric patients with asthma	mHealth	Telemedicine improved results compared to controls: improved self-management, improved patient satisfaction	Improved self-management, decreased medication use, increase in controlled asthma	Prospective study
Hsieh et al [44]	Insured adults (>20 years)	Patient portals	No control group (nonexperimental)	Not reported	Qualitative
Hutchesson et al [45]	Adult Australian women with a recent history of preeclampsia	mHealth	No statistically significant difference	No statistically significant difference	Pilot randomized controlled trial
Jiménez-Marrero et al [46]	Adult patients with chronic heart failure	Televideo	Telemedicine improved results compared to controls, decreased cost	Improved in at least one area: decreased incidence of heart failure	Randomized controlled trial
Katt et al [47]	180 patients with upper-extremity condition and 302 physicians	Telephone or televideo	Improved patient satisfaction	Not reported	Qualitative
Kobe et al [48]	Adult patients (52% men, mean age 62 years, 55.5% African American) of Duke University Health System with type 2 diabetes, poorly controlled hypertension, and on prescription hypertension and diabetes medication	Telephone or televideo	Telemedicine improved results compared to control	Improved in at least one area, improved annual rate eGFR^b^ decline	Secondary analysis of randomized controlled trial
Lai et al [49]	Adults with Parkinson disease (telehealth mean age 63 years, control mean age 70 years; 70% men, predominantly White)	Telemonitoring	Telemedicine improved results compared to control: improved compliance/adherence, health behaviors, and patient satisfaction	Not reported	Mixed methods
Lemelin et al [50]	Adult women (mean age 32 years) with gestational diabetes mellitus	Telecoaching	Improved patient satisfaction: telemedicine improved results compared to control	Identified other areas for intervention	Prospective and controlled clinical trial
Manning et al [51]	Adults from families with toddlers	Televideo	No statistically significant difference	Not reported	Mixed method quasiexperimental and longitudinal design
Marques et al [52]	Adult Valladolid University students (74% women, 67.5% aged 18-23 years)	mHealth	No control group (nonexperimental)	Not reported	Qualitative
Martins et al [53]	Adult patients (mean age 62 years, 50% women) with suspected acute strokes at a Brazil university hospital	mHealth	Telemedicine improved results compared to control	Improved in at least one area: decreased mortality, decreased intracranial hemorrhage	Prospective observational
McGillicuddy et al [54]	Adults (mean 51.5-52.1 years) with kidney transplants (majority men, African American)	mHealth	Telemedicine improved results compared to control	Improved in at least one area: reduction in mean tacrolimus trough coefficient of variation	Randomized controlled clinical trial
Mo et al [55]	Adult patients (51.7-53.5 years) with chronic heart failure (approximately 66% men)	Telephone or televideo	Telemedicine improved results compared to control: improved emotional support	Improved in at least one area: mental health inventory, quality of life	Open-label interventional study
Mustonen et al [56]	Adult patients (>45 years; mean age 65 years) with type 2 diabetes and coronary artery disease (approximately 40% women)	Telecoaching	No statistically significant difference	Not reported	Posttrial analysis of a randomized controlled trial
O’Shea et al [57]	Adults (77% men, mean age 61 years)	eHealth	Not reported	Not reported	Posttrial analysis of an acceptability and feasibility trial
Perri et al [58]	Adults (mean 55.4 years) from 14 counties in Florida (83% women, 73.9% White)	Telephone or televideo	Telemedicine improved results compared to control: decreased fat mass, improved self-management	Improved in at least one area: decreased body weight	Randomized clinical trial
Piera-Jiménez et al [59]	Adults (majority 50-70 years and men) from Spain, the Netherlands, and Taiwan	Telemonitoring	Telemedicine improved results compared to control	Improved in at least one area, improved quality of life	Financial randomized controlled trial
Press et al [60]	Adults (mean 54.5 years) with asthma or chronic obstructive pulmonary disease (majority Black women)	mHealth	Telemedicine improved results compared to control: improved self-management health behaviors	Increase in controlled asthma	Randomized controlled trial
Ramirez-Correa et al [61]	Adults (mean 39.9 years, 56% men)	Telemedicine self-testing	No control group (nonexperimental)	Not reported	Cross-sectional
Ronan et al [62]	Adults with cystic fibrosis involved in a study on an online Tai Chi intervention	Televideo	No statistically significant difference, improved health behaviors	Not reported	Qualitative analysis of a mixed methods randomized controlled feasibility study
Sacco et al [63]	Older adults (mean age 88.2 years), 59.8% women	Telephone or video	Improved patient satisfaction, improved emotion support	Not reported	Cross-sectional survey
Scheerman et al [64]	Adolescents (12-17 years) and mothers	Social media	Telemedicine improved results compared to control, improved health behaviors	Not reported	Cluster randomized controlled trial
Schrauben et al [65]	Adult Chronic Renal Insufficiency Cohort (CRIC) Study participants (mean age 68 years, eGFR 54 mL/min/1.73, 59% men)	mHealth	No control group (nonexperimental)	Not reported	Cross-sectional survey
Shareef et al [66]	Elderly and disabled people (average age 74.5 years, 59% women) in retirement homes and rehabilitation centers	Robotics or artificial intelligence	Improved companionship	Not reported	Experiment and follow-up survey
van Dijk et al [67]	Adult women (mean age 30 years), either less than 13 weeks pregnant or trying to become pregnant, and 36 men	mHealth	Improved compliance/adherence, improved health behaviors	Improved in at least one area, improved self-management	Randomized controlled trial

^a^mHealth: mobile health.

^b^eGFR: estimated glomerular filtration rate.

### Risk of Bias Within and Across Studies

Table 2 summarizes the quality indicators assessed for each article with the JHNEBP tool. The strength of evidence most frequently observed was level III followed by level I and level II. Nearly half of the articles reported strong-evidence studies that included both a control group and randomization; the next most common study type was nonexperimental (no control group) or qualitative, with the least frequent type being quasiexperimental (included a control group but no randomization). The quality of evidence most frequently observed was A (high quality), followed by B (good quality). The most common combination of strength and quality was III B, followed closely by I A, which speaks to both the strength and quality of evidence evaluated by this review. The III B combination highlights the number of qualitative studies with smaller samples or selection bias.

**Table 2:** Summary of quality assessments (N=46).

Evidence		Occurrence, n (%)
**Strength**		
	I (Experimental study or randomized controlled trial)	22 (48)
	III (Nonexperimental, qualitative)	17 (37)
	II (quasiexperimental)	7 (15)
**Quality**		
	A (High quality)	27 (59)
	B (Good quality)	17 (37)
	C (Low quality)	2 (4)

Many studies used geographically localized samples, which may limit the external validity of the results. Some studies focused only on one gender or race, speaking to the convenience sample or volunteer-basis of their design. Asking for volunteers in a technology-oriented experiment invites bias because the self-selection allows for those who are already technology-oriented or comfortable with technology to participate. This group as the intervention can skew the results because those already comfortable with technology will not experience the frustration experienced by those who are not comfortable with technology. This selection bias also limits the external validity of the results. A comprehensive list of bias, country of origin, sample size, strength, and quality of evidence identified for each study can be found in Multimedia Appendix 1.

### Thematic Analysis Based on Results of Individual Studies

During the analysis phase of the systematic review process, the reviewers recorded observations to identify instances of patient satisfaction, as well as both facilitators and barriers to the adoption of telemedicine. A thematic analysis was then performed to make sense of the observations [19]. Multiple instances of the same observation become a theme. A translation of observations to themes is provided in Multimedia Appendix 2. The summary of analysis is provided in Table 3, which lists the themes/observations from reviewers that correspond with the objective statement and sorts articles from the most recent to the oldest.

**Table 3:** Summary of thematic analysis for individual studies.

Authors	Patient satisfaction	Facilitators	Barriers
Ben-Arye et al [22]	Not reported	Technical literacy, availability of technology, past experience with technology	Availability of technology, confidentiality/security
Yu et al [25]	Strong satisfaction	Concerns adequately addressed, improved health behaviors, pandemic created acceptance of technology	Some patients prefer in-person consultations, decrease in patient-provider communication, technical literacy
Richards et al [24]	Strong satisfaction	Convenience of telemedicine, increased patient-provider communication, concerns adequately addressed, increased access	Not reported
Kurihara et al [23]	Not reported	Pandemic created acceptance of technology, past experience with technology	Some patients prefer in-person consultations, technical literacy
Alkirie et al [26]	Not reported	Technical literacy, past experience with technology, perceived usefulness, increased patient-provider communication, perceived ease of use	Technology needs further development, technical literacy
Ballin et al [27]	Not reported	Increased connectedness, self-management, flexibility, and access	Technology needs further development
Banbury et al [28]	Not reported	Enabled social interaction; decreased anxiety; increased connectedness, technical literacy, and access; televideo enables reading of body language; education; convenience of telemedicine	Health literacy, availability of technology, technical literacy
Barnett et al [29]	Not reported	Increased efficiency, access, and patient-provider communication, and improved standard of care	Technology needs further development, decrease in patient-provider communication, technical literacy, confidentiality/security
Batalik et al [30]	Not reported	Technical literacy, increased self-management, increased access, increased flexibility	Discomfort for wearable monitors, technical literacy, technology needs further development
Beller et al [31]	Not reported	Pandemic created acceptance of technology, availability of technology, fewer miles driven to appointment, convenience of telemedicine, faster initiation of treatment, decreased costs	Limits of reimbursement for telemedicine, some patients prefer in-person consultations, connectivity, technical literacy
Bernabe-Ortiz et al [32]	Not reported	Increased connectedness, increased adherence, improved health behaviors	Perceived lack of usefulness, lack of personal desire to get better, some patients prefer in-person consultations
Bilgrami et al [33]	Not reported	Pandemic created acceptance of technology	Not reported
Broers et al [34]	Strong satisfaction	Perceived usefulness, perceived ease of use, increased adherence	Decrease in quality of life after intervention
Cho et al [35]	Not reported	Increased adherence, increased self-management, increased weight loss, technical literacy	Technical literacy, availability of technology
Claes et al [36]	Not reported	Technical literacy, perceived ease of use	Technology needs further development
Coorey et al [37]	Not reported	Increased adherence, increased self-management	Lack of personal desire to get better, technology needs further development, technical literacy
Ding et al [38]	Not reported	Increased adherence, increased self-management	Technology needs further development, cost
Geramita et al [39]	Not reported	Long-term use may not be required to develop good habits	Cost, confidentiality/security, technology needs further development
Gong et al [40]	Not reported	Increased adherence, increased self-management	Not reported
Han et al [41]	Not reported	Pandemic created acceptance of technology, increased efficiency, increased self-management, increased access, availability of technology	Cost, technical literacy, interoperability, availability of technology
Harding et al [42]	Not reported	Not reported	Connectivity, confidentiality/security, technical literacy
Hsia et al [43]	Strong satisfaction	Increased quality of life, decreased emergency room visits, increased adherence, availability of technology, pandemic created acceptance of technology, perceived ease of use, convenience of telemedicine	Connectivity, technical literacy, cost, availability of technology
Hsieh et al [44]	Not reported	Health literacy, perceived usefulness, perceived ease of use	Some patients prefer in-person consultations, technical literacy, cost
Hutchesson et al [45]	Strong satisfaction	Increased self-management, perceived usefulness, perceived ease of use	Technology needs further development, perceived lack of usefulness
Jiménez-Marrero et al [46]	Not reported	Decreased costs, increased adherence, increased self-management	Cost
Katt et al [47]	Strong satisfaction	Convenience of telemedicine, pandemic created acceptance of technology, faster initiation of treatment, perceived ease of use	Some patients prefer in-person consultations, workflow issues for providers
Kobe et al [48]	Not reported	Not reported	Some patients prefer in-person consultations
Lai et al [49]	Strong satisfaction	Convenience of telemedicine, increased social support, increased self-management	Technology needs further development, connectivity, decrease in patient-provider communication, technical literacy
Lemelin et al [50]	Strong satisfaction	Education, increased social support	Not reported
Manning et al [51]	Not reported	Pandemic created acceptance of technology	Connectivity, availability of technology
Marquez et al [52]	Not reported	Past experience with technology, decreased costs, pandemic created acceptance of technology, faster initiation of treatment, increased access	Some patients prefer in-person consultations
Martins et al [53]	Not reported	Faster initiation of treatment, availability of technology, increased access	Lack of infrastructure, limits of reimbursement for telemedicine, connectivity, confidentiality/security
McGillicuddy et al [54]	Not reported	Increased social support, health literacy	Not reported
Mo et al [55]	Not reported	Increased quality of life, increased social support	Not reported
Mustonen et al [56]	Not reported	Decreased costs	Not reported
O’Shea et al [57]	Satisfaction	Increased self-management	Technical literacy, perceived lack of usefulness, technology needs further development
Perri et al [58]	Not reported	Increased weight loss, increased adherence, increased self-management	Not reported
Piera-Jiménez et al [59]	Not reported	Decreased costs, no significant difference in cost care	Cost
Press et al [60]	Not reported	Decreased costs, education, increased access	Availability of technology, technical literacy
Ramirez-Correa et al [61]	Not reported	Increased patient-provider communication, education, pandemic created acceptance of technology	Connectivity
Ronan et al [62]	Not reported	Convenience of telemedicine, pandemic created acceptance of technology, increased social support	Technical literacy, technology needs further development, availability of technology
Sacco et al [63]	Strong satisfaction	Increased social support, increased connectedness	Not reported
Scheerman et al [64]	Not reported	Increased social support, improved standard of care	Not reported
Schrauben et al [65]	Not reported	Health literacy, education	Technical literacy, health literacy, confidentiality/security
Shareef et al [66]	Not reported	Enabled social interaction, increased social support	Confidentiality/security, technical literacy, perceived lack of usefulness
van Dijk et al [67]	Not reported	Improved health behaviors, increased adherence	Not reported

Patient satisfaction was reported as “strong satisfaction” or “satisfaction” in 9 (20%) and 1 (2%) of the 46 studies, respectively, and 36 studies did not report any measure of patient satisfaction. No studies reported a decline in patient satisfaction as a result of using telemedicine as the intervention.

Twenty-five facilitator themes and seven individual observations were identified in the literature by the two reviewers. Only two studies did not identify facilitators. Facilitator themes are listed in Table 4.

**Table 4:** Facilitator themes and individual observations (N=132).

Themes/observations	References	Occurrence, n (%)
Increased self-management	[27,30,35,37,38,40,41,45,46,49,57,58]	12 (9.1)
Pandemic created acceptance of technology	[23,25,31,33,41,43,47,51,52,61,62]	11 (8.3)
Increased adherence	[32,34,35,37,38,40,43,46,58,67]	10 (7.6)
Increased access	[24,27-30,41,52,53,60]	9 (6.8)
Increased social support	[49,50,54,55,62-64,66]	8 (6.1)
Convenience of telemedicine	[24,28,31,43,47,49,62]	7 (5.3)
Perceived ease of use	[26,34,36,43-45,47]	7 (5.3)
Decreased costs	[31,46,52,56,59,60]	6 (4.5)
Education	[28,50,60,61,65]	5 (3.8)
Technical literacy	[22,26,30,35,36]	5 (3.8)
Availability of technology	[22,31,41,43,53]	5 (3.8)
Increased patient-provider communication	[24,26,29,61]	4 (3.0)
Faster initiation of treatment	[31,47,52,53]	4 (3.0)
Increased connectedness	[27,28,32,63]	4 (3.0)
Perceived usefulness	[26,34,44,45]	4 (3.0)
Past experience with technology	[22,23,26,52]	4 (3.0)
Health literacy	[44,54,65]	3 (2.3)
Improved health behaviors	[25,32,67]	3 (2.3)
Increased efficiency	[29,41]	2 (1.5)
Concerns adequately addressed	[24,25]	2 (1.5)
Enabled social interaction	[28,66]	2 (1.5)
Increased quality of life	[43,55]	2 (1.5)
Improved standard of care	[29,64]	2 (1.5)
Increased flexibility	[27,30]	2 (1.5)
Increased weight loss	[35,58]	2 (1.5)
Decreased anxiety	[28]	1 (0.8)
Increased technical literacy	[28]	1 (0.8)
Televideo enables reading of body language	[28]	1 (0.8)
Fewer miles driven to appointment	[31]	1 (0.8)
Long-term use may not be required to develop good habits	[39]	1 (0.8)
Decreased emergency room visits	[43]	1 (0.8)
No significant difference in cost of care	[59]	1 (0.8)
Not reported	[42,48]	2 (N/A^a^)

^a^N/A: not applicable.

The most commonly identified themes were increased self-management, acceptance of the technology from the pandemic, adherence to treatment protocols, access, and social support. For the 46 articles, these themes represent 38% of all 132 occurrences. Other themes included convenience of telemedicine and perceived ease of use, decreased cost, opportunity for education, technical literacy, availability of technology, an increase in patient-provider communication, faster initiation of treatment, increased connectedness, perceived usefulness, and past experience with technology. Health literacy and improved health behaviors were identified less frequently, and increased office efficiencies, medical concerns adequately addressed, enabled social interaction, increased quality of life, improved standard of care, increased flexibility, and increased weight loss were the least frequent themes identified. The following seven individual observations accounted for 5% of the total observations: decreased anxiety, increased technical literacy, televideo enabled reading of body language, fewer miles driven to appointment, long-term use may not be required to develop good habits, decreased emergency room visits, and no significant difference in cost of care.

Twelve themes and five individual observations were identified as barriers from the literature by the reviewers; 11 studies did not identify barriers (11%). Table 5 lists the themes and individual observations.

The most commonly listed barriers were technical literacy, technology needs further development, availability of technology, and patient preference, accounting for 55% of the total 86 occurrences. Cost, connectivity, and confidentiality/security were also identified, as well as health literacy, limits of reimbursement for telemedicine, and lack of personal desire to get better with less frequent occurrences (2 each). The remaining five observations made up a total of 6% of the total occurrences: decrease in quality of life after intervention, discomfort for wearing monitors, workflow issues for providers, lack of data infrastructure, and interoperability.

**Table 5:** Barrier themes and individual observations (N=86).

Themes/observations	References	Occurrence, n (%)
Technical literacy	[23,25,26,28-31,35,37,41-44,49,57,60,62,65,66]	19 (22)
Technology needs further development	[26,27,29,30,36-39,45,49,57,62]	12 (14)
Availability of technology	[22,28,35,41,43,51,60,62]	8 (9)
Cost	[38,39,41,43,44,46,59]	7 (8)
Connectivity	[31,42,43,49,51,53,61]	7 (8)
Confidentiality/security	[22,29,39,42,53,65,66]	7 (8)
Some patients prefer in-person consultations	[23,25,31,32,44,47,48,52]	8 (9)
Perceived lack of usefulness	[32,45,57,66]	4 (5)
Decrease in patient-provider communication	[25,29,49]	3 (3)
Health literacy	[28,65]	2 (2)
Limits of reimbursement for telemedicine	[31,53]	2 (2)
Lack of personal desire to get better	[32,37]	2 (2)
Decrease in quality of life after intervention	[34]	1 (1)
Discomfort for wearable monitors	[30]	1 (1)
Workflow issues for providers	[47]	1 (1)
Lack of infrastructure	[53]	1 (1)
Interoperability	[41]	1 (1)
Not reported	[24,33,40,50,54-56,58,63,64,67]	11 (N/A^a^)

^a^N/A: not applicable.

### Additional Analyses

#### Distribution of Publications by Country

Eighteen of the 46 studies (39%) were performed in North America, 11 (24%) were performed in Europe, 7 (15%) were performed in Asia, 5 (11%) were performed in Australia, 3 (7%) were performed in South America, and 2 (4%) were performed in multiple countries and continents.

#### Comparisons to a Control Group

Table 6 summarizes the themes and observations recorded for results as compared to the control group identified by the two reviewers. There is some overlap between this set of observations and medical outcomes; the latter represent clinical observations only, whereas the former are both clinical and administrative in nature. Ten themes and four individual observations were identified by the reviewers for a total of 66 occurrences in the literature. Eleven studies were nonexperimental in nature, which had no control group.

Eighteen of the studies demonstrated either a clinical or administrative improvement compared to the control group, whereas nine reported no statistically significant results from the control group. Both of these themes demonstrate the efficacy of the telemedicine modality. The remainder of the list in Table 6 demonstrates the specific improvements that occurred (multiple improvements occurred in multiple articles), including improved patient satisfaction, improved behaviors, improved compliance/adherence to treatment protocol, improved self-management of condition or disease, decreased fat mass, improved emotional support, improved companionship, and improved health literacy. The remainder were individual observations that combined accounted for 5% of the total observations: improved informational support, decreased cost, and increased quality of life. Only one article did not report a result as compared to the control group because it was a posttrial analysis and it did not address the control group.

**Table 6:** Themes and individual observations for studies with a control group comparison (N=66).

Themes/observations	References	Occurrence, n (%)
Telemedicine improved results compared to control	[28,32,35,38,40,41,43,46,48-50,53-55,58-60,64]	18 (27)
No statistically significant difference	[27,30,33,34,39,45,51,56,62]	9 (14)
Improved patient satisfaction	[24,25,43,47,49,50,63]	7 (11)
Improved health behaviors	[36,49,60,62,64,67]	6 (9)
Improved compliance/adherence	[22,38,40,41,49,67]	6 (9)
Improved self-management	[28,37,43,58,60]	5 (8)
Decreased fat mass	[27,32,35,58]	4 (6)
Improved emotional support	[28,55,63]	3 (5)
Improved companionship	[28,66]	2 (3)
Improved health literacy	[28,37]	2 (3)
Improved informational support	[28]	1 (2)
Decreased cost	[46]	1 (2)
Increased quality of life	[34]	1 (2)
Not reported	[57]	1 (2)
No control group (nonexperimental)	[23,26,29,31,37,41,42,44,52,61,65]	11 (N/A^a^)

^a^N/A: not applicable.

#### Medical Outcomes Commensurate With an Intervention

Table 7 summarizes the medical outcomes observed. Seven themes and nine individual observations were recorded commensurate with the adoption of telemedicine for a total of 30 occurrences. Twenty-eight studies did not report clinical outcomes.

Twelve studies reported 12 statistically significant improvements in clinical outcomes and three reported no statistically significant difference between modalities of care. Both of these themes demonstrated the efficacy of the telemedicine modality. The most commonly observed theme for medical outcomes was decreased body weight, followed by decreased fat mass, improved self-management, increase in controlled asthma, and increased quality of life. The following individual observations contributed to 30% of the total observations: reduction in blood pressure, reduction in mean tacrolimus trough coefficient of variation, improved annual rate of estimated glomerular filtration rate (eGFR) decline, decrease in medication use, decrease incidence of heart failure, decreased mortality, improved mental health inventory, decreased intracranial hemorrhage, and telemedicine identified other areas for intervention.

**Table 7:** Medical outcome themes and individual observations commensurate with adoption of the intervention/technology (N=30).

Themes/observations	References	Occurrence, n (%)
Improved in at least one area	[27,32,35,40,46,48,53-55,58,59,67]	12 (40)
No statistically significant difference	[30,33,45]	3 (10)
Decreased body weight	[32,35,58]	3 (10)
Decreased fat mass	[27,35]	2 (7)
Improved self-management	[43,67]	2 (7)
Increase in controlled asthma	[43,60]	2 (7)
Improved quality of life	[55,59]	2 (7)
Reductions in blood pressure	[40]	1 (3)
Reduction in mean tacrolimus trough coefficient of variation	[54]	1 (3)
Improved annual rate of eGFR^a^ decline	[48]	1 (3)
Decreased medication use	[43]	1 (3)
Decreased incidence of heart failure	[46]	1 (3)
Identified other areas for intervention	[50]	1 (3)
Decreased mortality	[53]	1 (3)
Improved mental health inventory	[55]	1 (3)
Decreased intracranial hemorrhage	[53]	1 (3)
Not reported	[22-26,28,29,31,34,36-39,41,42,44,47,49,51,52,56,57,61-66]	28 (N/A^b^)

^a^eGFR: estimated glomerular filtration rate.

^b^N/A: not applicable.

#### Interactions Between Observations

Interventions of mHealth resulted in seven occurrences of a result (clinical and administrative outcomes) and six occurrences of an improvement in at least one clinical outcome. The interventions with telephone or televideo resulted in four instances of improved patient satisfaction and a decrease in eGFR and weight loss. The interventions of eHealth resulted in very few instances of either clinical or administrative outcomes other than improved compliance and health behaviors.

## Discussion

### Principal Findings

Telemedicine is examined in countries worldwide, and it is clear that the COVID-19 pandemic caused a rapid adoption of this modality of medicine to ensure the viability of practices. A key issue for discussion is the differences in findings between this systematic review and another recent similar review [14]. This systematic review identified key facilitators and barriers, and further analyzed health outcomes. The other similar review identified themes of effectiveness but failed to meet the expectations for a systematic review in terms of medical outcomes [68]. Common themes between the two reviews were: rapid telemedicine expansion, education, improved access, convenience, and patient satisfaction.

### Summary of Evidence

This systematic review exercised a set Boolean search string in four common research databases to analyze 46 articles originating from five continents for themes of facilitators, barriers, and medical outcomes. Nearly 50% of the articles demonstrated the strongest evidence and nearly 60% demonstrated the highest quality of evidence. Various forms of telemedicine were examined: eHealth, mHealth, audio only, telemonitoring, telecoaching, telerehab, robotics or artificial intelligence, and televideo. Twenty-five facilitator themes and individual observations, 12 results themes and observations, and 7 medical outcome themes and observations were recorded and analyzed. Forty-one percent of barrier themes recorded either an improvement or no statistically significant improvement in results compared to the control group. Forty percent of the observations recorded an improvement in at least one medical outcome.

Health care administrators can focus on the findings demonstrating that implementation of telemedicine will increase self-management [27,30,35,37,38,40,41,45,46,49,57,58], adherence [32,34,35,37,38,40,43,46,58,67], access [24,27-30, 41,52,53,60], and social support [49,50,54,55,62-64,66]. Telemedicine is shown to be an effective modality of treatment [28,32,35,38,40,41,43,46,48-50,53-55,58-60,64] at a decreased cost [31,46,52,56,59,60]. Patients perceive the modality to be convenient and easy to use [26,34,36,43-45,47], and its implementation increases patient satisfaction [24,25,43, 47,49,50,63].

Health policymakers should focus on several barriers to increase the adoption of telemedicine. Because technical literacy, availability of technology, and connectivity are listed as the most often cited barriers, public programs should be offered to assist those with these difficulties. Technical literacy is often associated along age or socioeconomic lines, and researchers acknowledge the dearth of research in the area of how to overcome this obstacle [69]. However, community centers that provide access to computers, classes on computers, and a dedicated broadband connection can all contribute to solutions to these barriers.

A key similarity between the 2020 systematic review [14] and this review is the rapid expansion of telemedicine. Eleven articles analyzed in this review used a phrase similar to “the pandemic created an acceptance of telemedicine technology” [23,25,31,33,41,43,47,51,52,61,62]. A systematic review published in 2018 cited cost as the chief barrier to adoption, whereas this review only found cost as a barrier in 8% of all observations [5]. The COVID-19 pandemic forced acceptance of the technology and enabled providers to not focus so intently on the cost of its implementation.

### Limitations

This systematic review selected 46 articles for analysis from four commonly available research databases. A larger group for analysis could have yielded richer results. This review also only utilized two researchers to analyze the data; additional researchers could have identified additional themes. Selection bias was controlled through independent analysis of all articles by both reviewers followed by consensus meetings. Publication bias is the largest limitation because we were unable to query and analyze unpublished articles.

### Conclusion

The COVID-19 pandemic caused huge problems to deliver medicine traditionally. However, these problems created an environment that limited face-to-face medical encounters and fostered legislation to reimburse the telemedicine modality for broad and rapid adoption of telemedicine to expand the access of care beyond the physical walls of the clinic. Physicians should feel confident that the telemedicine modality will be reimbursed and will have very little effect on patient satisfaction. Health care administrators who have not already adopted telemedicine should feel confident in the technology; however, they should ensure that sufficient confidentiality and security measures are in place. Policymakers should enact legislation to remove or mitigate barriers such as availability of technology, technical literacy, and connectivity, as these are commonly referred to in the literature.

## Appendix

Multimedia Appendix 1 Sample size, bias, effect size, country of origin, and statistics used.

Multimedia Appendix 2 Observation-to-theme conversion for patient satisfaction as well as facilitators and barriers to adoption.

## Abbreviations

CARES: Coronavirus Aid, Relief, and Economic Security
eGFR: estimated glomerular filtration rate
JHNEBP: Johns Hopkins Nursing Evidence-Based Practice Rating Scale
MeSH: Medical Subject Headings
mHealth: mobile health
PICOS: participants, intervention, results, outcomes, study design
PRISMA: Preferred Reporting Items for Systematic Reviews and Meta-Analyses
