# Supplementary material for: Facilitators and Barriers to the Adoption of Telemedicine During the First Year of COVID-19: Systematic Review
Source: J Med Internet Res. 2022 Jan 4;24(1):e31752. doi: 10.2196/31752 (PMC8729874; doi:10.2196/31752)
Supplement: Multimedia Appendix 1 [file jmir_v24i1e31752_app1.docx]

**Multimedia Appendix 1:** Sample size, bias, effect size, country of origin, and statistics used.

| Authors | Sample Size | Bias within study | Effect Size | Country of Origin | Statistics Used | Strength of Evidence | Quality of Evidence |  |
| --- | --- | --- | --- | --- | --- | --- | --- | --- |
| Ben-Arye, E. et al [[19](#_ENREF_19)] | 56 | Small sample size, self-selection, online group had higher rates of breast cancer & lower rates of metastatic disease | Not reported | Israel | t-tests, measures of variability, fisher's exact test, univariate analysis, multivariate logistic regression model | II | B |  |
|  |  |  |  |  |  |  |  |  |
| Yu, J. et al [[22](#_ENREF_22)] | 95 | Small sample size, response bias, one facility | Not reported | US | Fischer's exact test, measures of central tendency | III | A |  |
| Richards, A.E. et al [[21](#_ENREF_21)] | 179 | Study conducted at one facility in one country, reporting bias - only 19% responded | Not reported | US | ANOVA F-test, chi-square & Fisher's exact | III | B |  |
| Kurihara, K. et al [[20](#_ENREF_20)] | 103 | Small sample size, only in Japan, only with PD patients, only one healthcare system | Not reported | Japan | chi-squared tests | III | B |  |
| Alkirie, L. et al [[23](#_ENREF_23)] | 268 | Inclusion of only two generations, U.S. only, short-term study, focused only on utilization of HCPP for patients, does not include other digital technology, survey design not inclusive of qualitative perspective | Significant interaction effect between perceived usefulness and generation on patients' experience β = -.152, t = 2.218, p < .05 AND a significant effect of generation on usage frequency (β = .243, t = 4.248, p < .01) | US | Partial least squares, convergent and discriminant validity, composite reliability and average variance, coefficients of determination of the endogenous constructs and predictive relevance | III | B |  |
| Ballin, M. et al [[24](#_ENREF_24)] | 77 | Included only obese participants, excluded those with disabilities, stroke, myocardial infarction, or other heart conditions, high bp | no significant effect on the primary outcome VAT from pre- to post-intervention, Cohen’s δ effect size [ES], 0.5, 95% CI, − 24 to 223, P = 0.11; SE decreased FM by 619 g (ES, 0.5, 95% CI, 22 to 1215, P < 0.05) compared to WE. | Sweden | normal distribution, central tendency, analyses of covariance, Cohen’s δ | I | A |  |
| Banbury, A. et al [[25](#_ENREF_25)] | 112 | Participants were self-selecting and non-randomized. Only Australian adults 50+ years of age | Effect size on intervention group: small | Australia | Descriptive statistics, univariate analysis, X2 with Fishers exact test for categorical comparisons, paired t-tests, central tendency, skewness and kurtosis coefficients, Cohen's d, | II | B |  |
| Barnett, A. et al [[26](#_ENREF_26)] | 28 | Non-randomized, non-experimental, only in Australia | Not reported | Australia | none | III | B |  |
| Batalik, L. et al [[27](#_ENREF_27)] | 56 | Patients from single hospital, single unit, single country, small sample size, short timeframe of study, willingness to participate bias | Not reported | Czech Republic | measures of central tendency, analysis of variance, Bonferroni post-hoc analysis | I | A |  |
| Beller, H. et al [[28](#_ENREF_28)] | 209 | U.S. only, non-randomized, participant willingness, self-selection, insurance factor | Not reported | US | measures of central tendency, t-tests, chi-square tests, multivariable logistic regression modeling | II | B |  |
| Bernabe-Ortiz, A. et al [[29](#_ENREF_29)] | 164 | Attrition rate, only Peruvian participants, no assessment of differential exposition to preventative interventions, recall & desirability bias | Not reported | Peru | measures of central tendency, Poisson regression models, linear mixed models, dose-response analysis | III | B |  |
| Bilgrami, Z. et al [[30](#_ENREF_30)] | 222 | High level of patient activation prior to intervention, patients were at referral centers (higher condition severity), level of care at in-person centers | Not reported | US | t-test, one-way analysis of variance, Pearson’s chi-square, linear regression model, percent changes in beta coefficient estimates | I | A |  |
| Broers, E.R. et al [[31](#_ENREF_31)] | 149 | High differences in education & reported depressive symptoms between control & intervention, only participants from two facilities, small & healthy sample participants, self-selection bias | Not reported | Spain, Netherlands | chi-squared tests, two-tailed t-tests, univariate & multivariate linear mixed model analysis, beta coefficients | I | A |  |
| Cho, S.M.J. et al [[32](#_ENREF_32)] | 160 | Sample size not as large as intended, variability in follow-up attendance, self-efficacy & proficiency bias, study length | Not reported | Korea | analysis of variance, t testing, linear mixed model, beta coefficient, measures of central tendency | I | A |  |
| Claes, J. et al [[33](#_ENREF_33)] | 120 | Selection bias, follow-up time. Technological errors, age difference in consenting/nonconsenting participants, non-equal representation of gender among participants | Moderate-to-vigorous intensity physical acvitiy = .42; diastolic blood pressure = -.49; cardiovascular risk score = −0.36 | Belgium & Ireland | measures of central tendency, y independent t test or Mann-Whitney U test, Chi-square, mixed-effects analysis of variance, Cohen d, Spearman correlation coefficients | I | A |  |
|  |  |  |  |  |  |  |  |  |
| Coorey, G. et al [[34](#_ENREF_34)] | 36 | Interviewee selection bias, self-report bias, small sample size, assumptions of researchers regarding unobservable mechanisms of intervention effects | Not reported | Australia | Not reported | III | B |  |
| Ding, H. et al [[35](#_ENREF_35)] | 184 | Self-reported compliance assessment, high number of participants discontinued treatment, older participants | Not reported | Australia | chi-square test, Fisher exact test, Wilcoxon signed-rank test, analysis of covariance model, Andersen-Gill model, | I | A |  |
| Geramita, E.M. et al [[36](#_ENREF_36)] | 105 | Outside variables impact nonadherence between end of RCT and this study, small sample size, some original participants of RCT not included | Not reported | US | t tests, χ2 tests, and McNemar tests, linear regression and logistic regression, | III | B |  |
| Gong, K. et al [[37](#_ENREF_37)] | 480 | Medication adherence measurement too subjective, follow-up period too short to evaluate long-term outcomes, study focused in China | Not reported | China | measures of central tendency, t-tests, chi-squared tests, nonparametric Wilcoxon signed-rank test | I | A |  |
| Han, J.K. et al [[38](#_ENREF_38)] | 526 | Differences in respondent size between surveys, overall sample size, unknown overlap between respondent response, digital divide, differences in pandemic impact, self-reported | Not reported | US | Categorical variables are reported as absolute numbers and percentages and responses to S1 and S2 were compared using the cx^2 test | III | B |  |
| Harding, R. et al [[39](#_ENREF_39)] | 149 providers / 837 patient assessments | Only a pilot study, only in three countries, selection bias in sites used, sample size of interviewees (27) | Not reported | India, Uganda, and Zimbabwe | Not reported | III | B |  |
| Hsia, B.C. et al [[40](#_ENREF_40)] | 39 | Small sample size, single arm (prospective), no historical data on patients, limited study time | For the visit 3 vs visit 1 comparison, the interaction effect was also significant (P ¼ .02) with OR of having well-controlled asthma being 13.8 among boys (95% CI, 2.56-74.4; P ¼ .003) and 1.00 among girls (95% CI, (0.26-3.92), P > .99). | US | Measures of central tendency, t tests, Wilcoxon, x^2 test, fisher's exact test, Power analysis: a one-sided McNemar’s test, generalized linear mixed model, Pearson’s correlations, linear mixed models | II | B |  |
| Hsieh, P.J. et al [[41](#_ENREF_41)] | 1160 | Not generalizable to all cloud-based service, younger respondent bias, participants were internet users, focused only in Taiwan | Not reported | Taiwan | structural equation modeling using partial least squares, multiple regression analysis, measures of reliability and validity | III | B |  |
| Hutchesson, M.J. et al [[42](#_ENREF_42)] | 31 | Not all women completed assessment, only in Australia, pilot study, limited data available on intervention, small sample size, short-term follow-up | Moderate effect sizes for total cholesterol (−0.58), LDL-C (−0.56), polyunsaturated fatty acid intake (0.51), sodium intake (−0.62) and resistance-based exercise (−0.75) suggesting potential for positive impacts of the intervention on health behaviors and CVD biomarkers | Australia | Measures of central tendency, Cohen’s d | I | B |  |
| Jiménez-Marrero, S. et al [[43](#_ENREF_43)] | 178 | Study conducted only in Spain, one center, small sample size, short follow-up period | Not reported | Spain | Measures of central tendency, Student’s t and x^2 tests, Kaplan Meier cumulative survivor function curves, log-rank tests, Cox proportional-hazards regression models, non-parametric tests | I | A |  |
| Katt, B. et al [[44](#_ENREF_44)] | 482 | U.S. only, small sample size, potentially not generalizable to other types of patients/providers | Not reported | US | Student t-tests were used to calculate P values for continuous data, and chi square tests were used for categorical data. Significance was established at a P value of < 0.05. All statistical analyses were performed using R Studio (Version 3.6.3; Vienna, Austria) | III | B |  |
| Kobe, E.A. et al [[45](#_ENREF_45)] | 281 | Only patients from one health system, study conducted only in the U.S. | not reported | US | descriptive statistics, 2-sample t tests, chi-squared tests, multivariable linear mixed model | III | B |  |
| Lai, B. et al [[46](#_ENREF_46)] | 20 | Small sample size, only PD patients, only in U.S., not randomized, control group patients were older level of support from telehealth coach not able to be appropriately measured, interviewer was also telecoach, lack of diversity within PD patients | Not reported | US | measures of central tendency, t-tests, Mann-Whitney U, chi-squared | II | C |  |
| Lemelin, A. et al [[47](#_ENREF_47)] | 161 | Not true randomized or blinded, only in Canada | Not reported | Canada | measures of central tendency, Tai's model | II | B |  |
| Manning, B.L. et al [[48](#_ENREF_48)] | 62 | Highly specific to audio, language. Families used own devices, not standardized, inconsistency with time of samples | small | US | nonparametric statistics, Mann-Whitney U tests, Wilcoxon signed-rank tests, measures of central tendency, Pearson's r, spearman correlations | II | A |  |
| Marqez, G. et al [[49](#_ENREF_49)] | 608 | Only students at one university, majority female respondents, one age group, one country | Not reported | Spain | Measures of central tendency, descriptive statistics | III | B |  |
| Martins, S.C.O. et al [[50](#_ENREF_50)] | 442 | Study performed in one stroke center, in one country, non-blinded neurologists | Not reported | Brazil | measures of central tendency, chi-squared, fisher exact tests, student t test, Mann-Whitney U test, kappa statistic | II | A |  |
| McGillicuddy, J.W. et al [[51](#_ENREF_51)] | 80 | Particpants were predominantly male, African American participants, study only in the United States, single transplant center, volunteer bias | Not reported | US | measures of central tendency, linear mixed models, descriptive statistics, independent univariate comparisons | I | A |  |
| Mo, Y. et al [[52](#_ENREF_52)] | 258 | Only in China, not double-blinded, single center, small sample | Not reported | China | measures of central tendency, continuous variables, student t test, chi squares test, linear regression analysis | II | A |  |
| Mustonen, E. et al [[53](#_ENREF_53)] | 1535 | Participants & healthcare professionals not blinded, participants had varying levels of participation in study activities, intervention too short | Not reported | Finland | Nonparametric bootstrapping, measures of central tendency | III | B |  |
| O'Shea, O. et al [[54](#_ENREF_54)] | 44 | Study conducted across two countries, but only in three clinics and predominantly older males | Not reported | Belgium & Ireland | Qualitative | III | B |  |
| Perri, M.G. et al [[55](#_ENREF_55)] | 445 | Predominantly white, female participants only from Florida, U.S.A. | Not reported | US | Measures of central tendency, Bayesian, approach, intent-to-treat estimate, percentage change analysis, cause mediational analysis | I | A |  |
| Piera-Jiménez, J. et al [[56](#_ENREF_56)] | 238 | Small sample size, difficulty in consolidating results from three cultural different countries, medication costs not considered. | Not reported | Spain, Netherlands, & Taiwan | Not reported | I | B |  |
| Press, V.G. et al [[57](#_ENREF_57)] | 118 | Majority Black, female participants, difference in baseline inhaler misuse between participants, single site study | Not reported | US | priori planned primary analysis, logistic regression, marginal probabilities, mixed-effects ordinal logit modeling | I | A |  |
|  |  |  |  |  |  |  |  |  |
| Ramirez-Correa, et al [[58](#_ENREF_58)] | 200 | Study only conducted in Brazil, younger mean age of participants may lead to higher acceptance of technology | Not reported | Brazil | descriptive analysis, reliability analysis, structural model analysis | III | B |  |
| Ronan, P. et al [[59](#_ENREF_59)] | 28 | Small sample size | Not reported | UK | Not reported | III | C |  |
| Sacco, G. et al [[60](#_ENREF_60)] | 132 | Small sample size, one country cultural considerations, not all confounding variables considered. | Not reported | France | Measures of central tendency, chi-square test, student t test | III | B |  |
| Scheerman, J.F.M. et al [[61](#_ENREF_61)] | 791 | Study focused in Iran, younger generation bias towards technology, culturally specific | extensive breakdown information based on each intervention and mediators | Iran | intention-to-treat principle, measures of central tendency, multilevel linear mixed modeling | I | A |  |
| Schrauben, S.J. et al [[62](#_ENREF_62)] | 932 | Additional confounding variables unable to be controlled for, ascertainment bias | Not reported | US | Poisson regression, measures of central tendency, and a qualitative content analysis of open-ended responses. | III | B |  |
| Shareef, M.A. et al [[63](#_ENREF_63)] | 179 | Only in Canada, cultural differences, variations in autonomous homecare systems | Not reported | Canada | Reliability testing, causal relationship structural modeling, CFA and construct validity | III | B |  |
|  |  |  |  |  |  |  |  |  |
|  |  |  |  |  |  |  |  |  |
| van Dijk, M.R. et al [[64](#_ENREF_64)] | 177 | Study performed in one center, small sample size, focused on primarily Dutch participants - cultural bias | Not reported | Netherlands | Multivariate linear regression analyses, bootstrapping | I | A |  |
|  |  |  |  |  |  |  |  |  |
|  |  |  |  |  |  |  |  |  |
