# Supplementary material for: Facilitators and Barriers to the Adoption of Telemedicine During the First Year of COVID-19: Systematic Review
Source: J Med Internet Res. 2022 Jan 4;24(1):e31752. doi: 10.2196/31752 (PMC8729874; doi:10.2196/31752)
Supplement: Multimedia Appendix 2 [file jmir_v24i1e31752_app2.docx]

**Multimedia Appendix 2:** Observation-to-theme conversion for patient satisfaction as well as facilitators and barriers to adoption.

| Authors | Patient Satisfaction | Patient Satisfaction Theme | Facilitators to adoption | Facilitators Theme | Barriers to adoption | Barriers Theme |  |
| --- | --- | --- | --- | --- | --- | --- | --- |
| Ben-Arye, E. et al [[19](#_ENREF_19)] | Not reported | Not reported | Familiarity with technology, access to technology, awareness of technology | Technical literacy | Barriers listed out. Geography, advanced age, culture barriers, socioeconomic status, lack of knowledge or awareness of available technologies, unsupportive caregiver, severity of illness, confidentiality & security, difficulty adhering to high standard of care, lack of emotional support. | Availability of technology |  |
|  |  |  |  | Availability of technology |  | Confidentiality / security |  |
|  |  |  |  | Past experience with technology |  |  |  |
| Yu, J. et al [[22](#_ENREF_22)] | Physicians (42% very satisfied or extremely satisfied, 40% neutral) & patients (84% very satisfied or extremely satisfied, 8% neutral) | Strong satisfaction | 94% of patients felt their concerns were addressed adequately during their telehealth visit. 72% of patients reported a desire to continue telehealth use after social distancing. | Concerns adequately addressed | Half of physicians reported feeling a need to schedule an in-person visit with patients to follow-up on the telehealth visit. Physicians may be worried about compromising the doctor-patient relationship as a result of telehealth replacing in-person encounters. 29% of physicians felt they adequately addressed their patients' concerns. Many physicians experience difficulties in using technology and may be concerned about their patients experiencing difficulties. | Some patients prefer in-person |  |
|  |  |  |  | Improved health behaviors |  | Decrease in patient-provider communication |  |
|  |  |  |  | Pandemic created acceptance of technology |  | Technical literacy |  |
| Richards, A.E. et al [[21](#_ENREF_21)] | Satisfaction rates over 92% | Strong satisfaction | Based on literature and assumption, high satisfaction likely linked to convenience and accessibility of telehealth appointments. Participants reported that they felt their provider showed concern for their questions and concerns, properly explained care, included patients in care decisions, and discussed proposed treatments | Convenience of telemedicine | Not reported | Not reported |  |
|  |  |  |  | Increased patient-provider communication |  |  |  |
|  |  |  |  | Concerns adequately addressed |  |  |  |
|  |  |  |  | Increased access |  |  |  |
| Kurihara, K. et al [[20](#_ENREF_20)] | Not reported | Not reported | COVID-19 pandemic created a state of emergency encouraging the utilization of telemedicine. Participants who responded that they had a positive attitude towards telemedicine were more likely to be experienced in using credit cards and smartphones. | Pandemic created acceptance of technology | Participants who responded that they had a negative attitude towards telemedicine were more likely to be inexperienced in using credit cards and smartphones. | Some patients prefer in-person |  |
|  |  |  |  | Past experience with technology |  | Technical literacy |  |
| Alkirie, L. et al [[23](#_ENREF_23)] | Not reported | Not reported | Technology training for Generation X participants allow them to become more comfortable utilizing technology, Millennials are comfortable with technology and therefore questioned perceived ease of use comparatively less than Gen X. Inclusion of generational difference considerations during technology design to minimize the impact of the digital divide, perceived usefulness of technology, perceived ease of use, technology training, effective patient-provider communication | Technical literacy | Gen X participants highly valued the usefulness of the technology and desired to see concrete positive results. Gen X participant desired more transparent, immediate, and actionable communication from providers. Generational barriers between tech designers and utilizers, lack of communication, lack of exposure, lack of training | Technology needs further development |  |
|  |  |  |  | Past experience with technology |  | Technical literacy |  |
|  |  |  |  | Perceived usefulness |  |  |  |
|  |  |  |  | Increased patient-provider communication |  |  |  |
|  |  |  |  | Perceived ease of use |  |  |  |
| Ballin, M. et al [[24](#_ENREF_24)] | Not reported | Not reported | Web-based exercise program included a component which connected them with "digital training peers" to promote feelings of relatedness. Web-based exercise program promoted feelings of autonomy by allowing participants to choose when to work out and their level of difficulty. Increased access, freedom and flexibility, individualized/customizable | Increased connectedness | The intervention was not initiated immediately after the participants were randomized, likely impacting effect size. Interventions not conducted at the same time. Potentially lower adherence in the web-based group during a longer study length. | Technology needs further development |  |
|  |  |  |  | Increased self-management |  |  |  |
|  |  |  |  | Increased flexibility |  |  |  |
|  |  |  |  | Increased access |  |  |  |
| Banbury, A. et al [[25](#_ENREF_25)] | Not reported | Not reported | Videoconferencing allowed those individuals living alone or with mobility difficulties to engage socially. In video conference groups with higher adherence rates. Convenience of the technology and social engagement were reported as benefits. Participants reported program increased their experience and confident with information technology resulting in increased access to support and healthcare information. One individual reported purchasing a computer as a result of her participation in the informational videoconferences, increasing her access to health information. Participants reported improved knowledge of using and understanding peripherals by discussing among peers and bringing their results to discuss with their providers. | Enabled social interaction | Some participants missed their video conference due to illness, caring responsibilities, or medical appointments. Women living with husbands reported the task of using the computer was typically given to their husband. Participant adherence was lower among individuals who reported a higher number of telehealth peripherals and less confidence in navigating their healthcare. Participants were provided technology and instruction on its use, however, patients may not have access to technology or skills to use it. | Health literacy |  |
|  |  |  | Videoconferencing a preferred alternative for those suffering from anxiety. | Decreased anxiety |  | Availability of technology |  |
|  |  |  | group members reported feeling more emotionally supported | Increased connectedness |  | Technical literacy |  |
|  |  |  | Participants reported program increased their experience and confident with information technology | Increased technical literacy |  |  |  |
|  |  |  |  | Increased access |  |  |  |
|  |  |  | Video conferencing preferred over standard conference call as it allowed participants to feel connected and interact using body language/facial expressions. | Tele-video enables reading of body language |  |  |  |
|  |  |  | Increased access to information | Education |  |  |  |
|  |  |  | Convenience of the technology and social engagement were reported as benefits. | Convenience of telemedicine |  |  |  |
| Barnett, A. et al [[26](#_ENREF_26)] | N/A | Not reported | Use of chatbots perceived as increasing efficiency of care, improving physician or service referrals. Additionally participants highlighted the potential for use in areas of triaging, facilitation of online interventions, and use in collaboration with human counselling to allow counselors to focus more on patients. Participants reported benefits including the minimization of human error, improvements in standard of care, increased access to knowledge, and the potential for future recommendations based on the synthesis of information from chatbots to create a more effective counseling program/practice. Improvements in efficiency, supplemental to human care i.e., hybrid system, minimization of human error, increased access to information/expertise; perceived increase in privacy/security. | Increased efficiency | Participants questioned whether or not they were communicating with a human or chat bot, impacting their perception of their conversation and interaction given past experiences with chatbots. Participants expressed concern for using chatbots in place of human counselling due to a lack of sophistication, emotional intelligence, and empathy from technology. Lack of participant willingness to share information if they believed they were communicating with a chatbot. Patient perception; lack of AI/chatbot sophistication, emotional intelligence, empathy; impersonal experience; discomfort; | Technology needs further development |  |
|  |  |  |  | Increased access |  | Decrease in patient-provider communication |  |
|  |  |  |  | Increased patient-provider communication |  | Technical literacy |  |
|  |  |  |  | Improved standard of care |  | Confidentiality / security |  |
| Batalik, L. et al [[27](#_ENREF_27)] | Not reported | Not reported | Older participants did not experience difficulty utilizing more complex technology. Motivational guidance and feedback allow for patients to engage in the training program from home. Participating from home allowed patients to adapt the program to suit their needs and lifestyle. Increased access, feedback mechanisms, adaptable to needs/lifestyle, | Technical literacy | Participants reported discomfort of wearable technology during exercise. Study experienced technological issues including inability to use software, poor internet connectivity, & low battery. Due to a lack of device battery, patients discontinued participation in physical activity. Difficulty experienced by researchers in conducting telephone calls due to lack of participant availability. | Discomfort for wearable monitors |  |
|  |  |  |  | Increased self-management |  | Technical literacy |  |
|  |  |  |  | Increased access |  | Technology needs further development |  |
|  |  |  |  | Increased flexibility |  |  |  |
| Beller, H. et al [[28](#_ENREF_28)] | Not reported | Not reported | High willingness among participants to engage in a virtual visit, possibly due to shifting attitudes towards telemedicine due to the pandemic. screening to ensure patients have access to technology & internet connection, patient education on virtual visit technology prior to appointment, reduced cost of transportation, faster initiation of treatment | Pandemic created acceptance of technology | Participants with commercial insurance were more likely to complete a virtual visit than those with public insurance. Some patients indicated that they had a preference of in-person appointment over virtual visit, expressed a lack of comfort with technology and camera capabilities. Failure of virtual visit appointment completion occurred due to no-show participants, difficulty connecting to the virtua platform, and clerical errors (scheduling the wrong patients for virtual visits). Discomfort with technology, lack of camera capabilities, lack of internet connectivity, lack of adherence, audio issues, administrative scheduling issues, lack of available interpreter | Limits of reimbursement for telemedicine |  |
|  |  |  |  | Availability of technology |  | Some patients prefer in-person |  |
|  |  |  |  | Fewer miles driven to appointment |  | Connectivity |  |
|  |  |  |  | Convenience of telemedicine |  | Technical literacy |  |
|  |  |  |  | Faster initiation of treatment |  |  |  |
|  |  |  |  | Decreased costs |  |  |  |
| Bernabe-Ortiz, A. et al [[29](#_ENREF_29)] | Not reported | Not reported | Participants who received motivational interview calls experienced the greatest reductions in body weight and BMI. Regular communication i.e., motivational interviewing calls and text messages improve retention of healthy behaviors and long-term impact. booster appointments, | Increased connectedness | Without inclusion of follow-up communication, participants are less likely to be engaged, motivated, and are more likely to decrease adherence than in-person participants. Lack of adherence, lack of motivation | Perceived lack of usefulness |  |
|  |  |  |  | Increased adherence |  | Lack of personal desire to get better |  |
|  |  |  |  | Improved health behaviors |  | Some patients prefer in-person |  |
| Bilgrami, Z. et al [[30](#_ENREF_30)] | Not reported | Not reported | TELE-IBD intervention is feasible and agreeable for the patients | Pandemic created acceptance of technology | Heterogeneity of telemedicine interventions. Participants in population had less room for improvements in Patient Activation and Self-Efficacy. Remote monitoring text messaging did not improve self-efficacy or patient activation compared to traditional care in IBD referral centers. | Not reported |  |
| Broers, E.R. et al [[31](#_ENREF_31)] | Patients self-reported to be overall satisfied with intervention | Strong satisfaction | Patients in the intervention group that received the behavioral program were satisfied and perceived the program as useful and ease to use. Participants reported not feeling any social pressure to utilize the devices. Patients perceived the program as safe because they were being monitored by health professionals. Participants had a higher than usual adherence rate, likely due to the participants not feeling as though the program was demanding | Perceived usefulness | Perceived quality of life decreased in the intervention group after the cessation of the behavioral intervention due to a lack of support. Some differences in control v. intervention group baseline, may demonstrate that certain subgroups of patients would be more open than others. | Decrease in quality of life after intervention |  |
|  |  |  |  | Perceived ease of use |  |  |  |
|  |  |  |  | Increased adherence |  |  |  |
| Cho, S.M.J. et al [[32](#_ENREF_32)] | Not reported | Not reported | More frequent utilization of the mHealth intervention resulted in a greater reduction in body weight. Digital literacy, willingness to modify lifestyle | Increased adherence | Lack of digital literacy, physiological and socioeconomic background | Technical literacy |  |
|  |  |  |  | Increased self-management |  | Availability of technology |  |
|  |  |  |  | Increased weight loss |  |  |  |
|  |  |  |  | Technical literacy |  |  |  |
| Claes, J. et al [[33](#_ENREF_33)] | Not reported | Not reported | The participants found the program easy to use. | Technical literacy | Usage of the online system declined over time likely due to the weakness of the heart rate tracker and the limited interactive activities. Due to software issues at the beginning of the intervention period requiring updates, participants may have been frustrated and their perception of the program negatively impacted. lack of validated accelerometry algorithms for wrist-worn devices, adherence, software problems, frustration in utilizing technology | Technology needs further development |  |
|  |  |  |  | Perceived ease of use |  |  |  |
| Coorey, G. et al [[34](#_ENREF_34)] | Not reported | Not reported | Participants utilized self-monitoring after receiving updated information to the application regarding their risk profile. Participants were trusting of and motivated to action by the prescriptive health information provided by the intervention. Participant’s confidence in participating in their healthcare encounters and knowledge of prescription medication confidence increased when provided with the EHR medication list and consumer drug information. Personalized goal setting and behavior tracking increased participant awareness of the implications of health behaviors and increased motivation. Some participants who were satisfied with previous doctor-patient information and encounters utilized the new EHR resources to improve understanding of and adherence to treatment (found particularly in patients who identified as being responsible for their health in collaboration with the provider). Participant response to the intervention was enhanced where the provider's recommendations & advice coincided with the content provided in the intervention. | Increased adherence | Personal illness and the necessary attention required to attend to imminent health problems as opposed to prevention of future health problems a barrier to prevention techniques and programs. Participants with concerns regarding the legitimacy of risk data or an underestimation of the disease risk did not utilize EHR information. Participants who perceived the information was too burdensome resulted in disengagement. Participants reported frustration and contempt for being reminded to do "what they either already knew or should be doing". Some participants who were satisfied with previous doctor-patient information and encounters were less likely to utilize the EHR features. For participants who reported themselves as being frequent application users, there was a lower frequency of use as a result of the intervention's functions and interface not meeting expectations compared to other devices or software. Participants with lower technology access, utilization, and literacy reported non-use of the intervention. | Lack of personal desire to get better |  |
|  |  |  |  | Increased self-management |  | Technology needs further development |  |
|  |  |  |  |  |  | Technical literacy |  |
| Ding, H. et al [[35](#_ENREF_35)] | Not reported | Not reported | Compliance of participants was high likely due to the use of reminder alerts and call center contact. The interactive reminders increased participant engagement as it allowed the nurses to use the reminders as teaching moments and reinforce self-management behaviors. Monitoring of patients may increase compliance through a surveillance effect. Reliable technology, positive user experience, | Increased adherence | Technical issues in the beginning of the trial may have motivated some participants to withdraw. Technical issues increased support costs. , perceived difficulty of using technology, | Technology needs further development |  |
|  |  |  |  | Increased self-management |  | Cost |  |
| Geramita, E.M. et al [[36](#_ENREF_36)] | Not reported | Not reported | Even for participants who discontinued use, the facilitation of habit development of healthy behaviors may not require long-term utilization of the intervention. Utilization in combination with other interventions, motivation, clinician feedback on technology use | Long-term use may not be required to develop good habits | Long term adherence in utilizing the program was not sustained. Patients may discontinue use of mobile health interventions if they do not feel they are useful or they no longer need them. There was no long-term funding in the intervention for follow-up support and therefore participants did not receive continued technical support. Perceived ineffective nature of technology, changing needs of patient, cost, privacy and health information security, app complexity | Cost |  |
|  |  |  |  |  |  | Confidentiality / security |  |
|  |  |  |  |  |  | Technology needs further development |  |
| Gong, K. et al [[37](#_ENREF_37)] | Not reported | Not reported | Participants were able to successfully improve hypertension management utilizing the application, thereby supporting that the application improved medication adherence. Reminders from mHealth application, education | Increased adherence | Not reported | Not reported |  |
|  |  |  |  | Increased self-management |  |  |  |
| Han, J.K. et al [[38](#_ENREF_38)] | Not reported | Not reported | Majority of participants reported that usage of digital tools, wearables, and smart devices and video-telehealth usage had increased since the start of the pandemic. The top three perceived benefits of digital health, as reported by respondents, are improvements in patient morbidity and mortality, improvements in workflow and efficiency, increased patient involvement and empowerment in their healthcare & treatment, and increased access to care. An increase in adoption on both the patient and provider side has increased frequency and availability of telehealth. Prescription of a digital tool, software, application, program, etc. by a provider. Increases in funding for telehealth technologies will increase their availability in rural and underserved areas. Benefit of remote care, increase in provider adoption, younger patients with no comorbidities, need for continued care during pandemic | Pandemic created acceptance of technology | Lack of workplace infrastructure or support for digital health was reported as a barrier by the majority of participants. Respondents listed a need for "workplace digital health infrastructure and support" as the primary barrier to the adoption of digital health. The need for clinical trials showing efficacy and/or safety was the considered the second most important barrier to adoption of digital health. The third most important barrier is the need for interoperability with electronic medical records. An additional perceived barrier is the need for reimbursement structure. Barriers that need to be addressed include considerations for those with cognitive impairments, language barriers, and the inadequacy of visual inspections. cost of mobile health devices, lack of access to technology, no internet connectivity, language barriers, cognitive impairments, inadequacy of visual inspections, | Cost |  |
|  |  |  |  | Increased efficiency |  | Technical literacy |  |
|  |  |  |  | Increased self-management |  | Interoperability |  |
|  |  |  |  | Increased access |  | Availability of technology |  |
|  |  |  |  | Availability of technology |  |  |  |
| Harding, R. et al [[39](#_ENREF_39)] | Not reported | Not reported | Not reported | Not reported | A lack of internet connectivity in some areas where the study was conducted and a lack of confirmation for message/info uploads created communication challenges. Some patients were disappointed with the intervention based on their expectations that they would receive treatment more quickly. The time limitations of training made the initial use of the app difficult. Privacy concern, lack of internet connectivity, lack of literacy, time required for training | Connectivity |  |
|  |  |  |  |  |  | Confidentiality / security |  |
|  |  |  |  |  |  | Technical literacy |  |
| Hsia, B.C. et al [[40](#_ENREF_40)] | Satisfaction with the application was high, as indicated by the mean CSQ-8 score of 30 out of the total score of 32 at each visit | Strong satisfaction | The intervention improved asthma control in male participants, improved participant perceived quality of life, and had a high overall satisfaction rate for the application usage. Overall acceptance among adolescents of asthma management applications. Benefits of cost reduction from utilizing the application to manage asthma when compared to cost of emergency department visits. Use of gamification in designing the application improve adherence and engagement. Engaging and educational content, text-messaging based platforms, user-centered design, access to internet connectivity, access to technology devices, convenience, ease of use, continued care during pandemic, | Increased quality of life | Participants accessed the platform only during office visits, access is reliant on transportation, availability, and cost. Lack of internet connectivity, lack of access to technology | Connectivity |  |
|  |  |  |  | Decreased Emergency Room visits |  | Technical literacy |  |
|  |  |  |  | Increased adherence |  | Cost |  |
|  |  |  |  | Availability of technology |  | Availability of technology |  |
|  |  |  |  | Pandemic created acceptance of technology |  |  |  |
|  |  |  |  | Perceived ease of use |  |  |  |
|  |  |  |  | Convenience of telemedicine |  |  |  |
| Hsieh, P.J. et al [[41](#_ENREF_41)] | Not reported | Not reported | Study supports that health literacy is a factor for measuring individuals' intentions to use health information technology. Participants who believed they were more susceptible to health complications were more likely to desire knowing about their health information so they can better manage and confirm health status. Cues to action such as symptoms of disease, media promotion of health, physician recommendations, and family members with health problems increase intentions to use health passbook technology. perceived ease of use, perceived usefulness, self-efficacy, high degree of susceptibility, high severity | Health literacy | Patients who perceive a higher degree of severity regarding their health are more likely to use in-person services than they are to adopt health IT. Patients who perceive the technology will require a significant amount of time, money, or effort to use are less likely to use it. Perceived barriers, cost, perceived difficulty of use | Some patients prefer in-person |  |
|  |  |  |  | Perceived usefulness |  | Technical literacy |  |
|  |  |  |  | Perceived ease of use |  | Cost |  |
| Hutchesson, M.J. et al [[42](#_ENREF_42)] | Many participants self-reported high levels of satisfaction with the intervention | Strong satisfaction | Participants reported high levels of usability, appropriateness, and satisfaction with the program. Email newsletters had high levels of engagement and reported ease of use and appropriateness. Goal setting, action planning, self-monitoring, feedback, perceived usefulness, | Increased self-management | Participants utilized the goal setting and tracking portions of the program much less with lower levels of satisfaction due to the lack of perceived usefulness importance, and the perceived time required to utilize the program components. Perceived time commitment, perceived lack of usefulness | Technology needs further development |  |
|  |  |  |  | Perceived usefulness |  | Perceived lack of usefulness |  |
|  |  |  |  | Perceived ease of use |  |  |  |
| Jiménez-Marrero, S. et al [[43](#_ENREF_43)] | Not reported | Not reported | Participants in the telemedicine group had lower risks of heart failure related and all-cause hospitalizations. Telemedicine was associated with lower costs fror heart failure care compared to the control group. High levels of adherence, reduced costs. Telemedicine allowed for daily remote monitoring of patients, increase the number of reminders and prompts patients received to follow recommendations, receive evaluations, and record their health information. | Decreased costs | Telemedicine was associated with higher costs of ambulatory care. | Cost |  |
|  |  |  |  | Increased adherence |  |  |  |
|  |  |  |  | Increased self-management |  |  |  |
| Katt, B. et al [[44](#_ENREF_44)] | Patients generally felt very satisfied (80%) with their encounter. Perceived convenience, no wait time, comfort of home, continued care during pandemic, lower cost | Strong satisfaction | Majority of patients found the telehealth visit participation was easy. New patients were overall more satisfied with the telehealth visit compared to follow-up patients. Telehealth preferred by some patients because of convenience, easy, and time saving benefits. No "wait times". Patients more accepting of the option because they saw it as a beneficial option during the pandemic. | Convenience of telemedicine | Patient perception that in-office visits have a higher quality of care. | Some patients prefer in-person |  |
|  |  |  |  | Pandemic created acceptance of technology | Physicians were less satisfied with the use of telemedicine, particularly when evaluating a new patient. | Workflow issues for providers |  |
|  |  |  |  | Faster initiation of treatment |  |  |  |
|  |  |  |  | Perceived ease of use |  |  |  |
| Kobe, E.A. et al [[45](#_ENREF_45)] | Not reported | Not reported | Not reported | Not reported | Increased telehealth usage may result in patient fatigue, disengagement, and poor health outcomes | Some patients prefer in-person |  |
|  |  |  |  |  |  |  |  |
| Lai, B. et al [[46](#_ENREF_46)] | Reported "overtly positive", but only qualitative information | Strong satisfaction | Participants in the telehealth group reported that the convenience of the program and monitoring and support feature made them feel accountable and motivated. Their confidence in exercise and using the technology was increased by the personalized nature of their telehealth coaching. | Convenience of telemedicine | Participants reported that the technology usage required a "learning curve" and internet connectivity issues were frequent and negatively impacted communication between participants and their coaches. Technology difficulties caused participant frustration and discontinuation of the program. | Technology needs further development |  |
|  |  |  |  | Increased social support |  | Connectivity |  |
|  |  |  |  | Increased self-management |  | Decrease in patient-provider communication |  |
|  |  |  |  |  |  | Technical literacy |  |
| Lemelin, A. et al [[47](#_ENREF_47)] | Satisfaction with care: 8.9/10 in intervention v. 8.5/10 in control. Satisfaction with educational support: 9.0/10 in intervention, 8.5/10 in control | Strong satisfaction | Participants in the telehealth group reported greater overall satisfaction and greater educational support satisfaction. | Education | Not reported | Not reported |  |
|  |  |  |  | Increased social support |  |  |  |
| Manning, B.L. et al [[48](#_ENREF_48)] | Not reported | Not reported | Families may not have in-person access to child language assessments or not feel comfortable in a research environment. COVID-19 pandemic increasing the need for validated tele practice measures. | Pandemic created acceptance of technology | Background noise and poor internet connectivity resulted in poor audio quality. | Connectivity |  |
|  |  |  |  |  |  | Availability of technology |  |
| Marqez, G. et al [[49](#_ENREF_49)] | Not reported | Not reported | Over 79% of respondents who had used mental health applications in the past were open to using them in the future. Respondents reported that their reasons for preference of mobile applications over consultations included associated cost of care, stigma, lack of knowledge of mental health, previous negative experiences, privacy & security, timesaving, social isolation required by the pandemic's lockdown measures, and distance/lack of access. | Past experience with technology | 36.8% of respondents who had not previously used mental health applications reported that they were doubtful or would not use them in the future. Of participants regarded as "severe", 55% said they would not prefer to use mental health applications. Respondents said they were not aware that mental health applications existed. | Some patients prefer in-person |  |
|  |  |  |  | Decreased costs |  |  |  |
|  |  |  |  | Pandemic created acceptance of technology |  |  |  |
|  |  |  |  | Faster initiation of treatment |  |  |  |
|  |  |  |  | Increased access |  |  |  |
| Martins, S.C.O. et al [[50](#_ENREF_50)] | Not reported | Not reported | High level of agreement between remote decision vs. in-person assessment. 27-minute reduction in door-to-needle time improving workflow. Increase in mobile/smartphone ownership across Brazil. | Faster initiation of treatment | Poor infrastructure and low funding of telemedicine limit usage. Acceptance of poor telecommunication may result in decreased clinical outcomes, and limited patient safety & privacy. | Lack of infrastructure |  |
|  |  |  |  | Availability of technology |  | Limits of reimbursement for telemedicine |  |
|  |  |  |  | Increased access |  | Connectivity |  |
|  |  |  |  |  |  | Confidentiality / security |  |
| McGillicuddy, J.W. et al [[51](#_ENREF_51)] | Not reported | Not reported | Participants with a spouse/significant other, above average self-efficacy, and above-average health literacy were more likely to experience success with the intervention. | Increased social support | Not reported | Not reported |  |
|  |  |  |  | Health literacy |  |  |  |
| Mo, Y. et al [[52](#_ENREF_52)] | Not reported | Not reported | Improved quality of life | Increased quality of life | Not reported | Not reported |  |
|  |  |  |  | Increased social support |  |  |  |
|  |  |  |  |  |  |  |  |
| Mustonen, E. et al [[53](#_ENREF_53)] | Not reported | Not reported | Decrease in cost of service, but not significant | Decreased costs | Not reported | Not reported |  |
| O'Shea, O. et al [[54](#_ENREF_54)] | Not quantitatively measured, but participants reported sense of satisfaction from engagement with PATHway system | Satisfaction | Participants engaging with PATHway system found text messaging motivational. The flexible nature of the programming combined with a personalized approach increased engagement. Participants felt the wearable wristband was motivational and helped with real-time self-monitoring. Technical support helped to assist with system issues. | Increased self-management | Lower engagement with some of the technology programming was due to a lack of awareness, lack of perceived need or appeal of program. Participant reported the online exercise game was unappealing and felt a personal disconnect between computers and exercise. Participants felt there were too many steps to using the program and too much equipment associated with it. Lack of powersockets, took too much electrical power, not feasible for self-setup. Participants reported frustration with the technology, sometimes choosing to not exercise if their technical problems took too long or their wearable wristband did not work. Lack of technology literacy was identified as a barrier. | Technical literacy |  |
|  |  |  |  |  |  | Perceived lack of usefulness |  |
|  |  |  |  |  |  | Technology needs further development |  |
| Perri, M.G. et al [[55](#_ENREF_55)] | Not reported | Not reported | Extended care via individual telephone counseling resulted in improved long-term weight loss management. Individual telephone counseling increased the number of days which participants achieved their goals and increased self-monitoring frequency. | Increased weight loss | Not reported | Not reported |  |
|  |  |  |  | Increased adherence |  |  |  |
|  |  |  |  | Increased self-management |  |  |  |
| Piera-Jiménez, J. et al [[56](#_ENREF_56)] | Not reported | Not reported | While the intervention was less effective in Spain, its cost threshold is reasonable. | Decreased costs | Intervention too costly with no enough cost-effectiveness in Netherlands and Taiwan. Cost effectiveness is a major factor for implementation of telemedicine | Cost |  |
|  |  |  |  | No significant difference in cost of care |  |  |  |
| Press, V.G. et al [[57](#_ENREF_57)] | Not reported | Not reported | Virtual intervention is associated with lower costs and increased access in terms of education delivery to patients. Virtual intervention allows patients to repeat training and education as needed. | Decreased costs | Interventions relying on technology are not feasible in all areas and for all patients | Availability of technology |  |
|  |  |  |  | Education |  | Technical literacy |  |
|  |  |  |  | Increased access |  |  |  |
| Ramirez-Correa, et al [[58](#_ENREF_58)] | Not reported | Not reported | Promotion of patient and provider engagement with technologies. Proper communication of technology benefits. | Increased patient-provider communication | Heterogeneous implementation of telemedicine across various countries. | Connectivity |  |
|  |  |  |  | Education |  |  |  |
|  |  |  |  | Pandemic created acceptance of technology |  |  |  |
| Ronan, P. et al [[59](#_ENREF_59)] | Not reported | Not reported | Participants noted the benefit of how convenient the home lessons were and that it decreased risk of infection. Practice partners or familial support increased enjoyment. | Convenience of telemedicine | Several participants experienced difficulties with technology. Withdrawal was higher in the internet based group. Participant concerns over feasibility for young children given outside distractions. Some participants experienced difficulty in seeing their instructor or positioning themselves and the technology so the instructor could see them. Teenage participants were more likely to skip classes. Some participants could not use the DVD because they did not own a DVD player. | Technical literacy |  |
|  |  |  |  | Pandemic created acceptance of technology |  | Technology needs further development |  |
|  |  |  |  | Increased social support |  | Availability of technology |  |
| Sacco, G. et al [[60](#_ENREF_60)] | (46/73, 87%, with telephone calls versus 47/59, 89%, with video calls | Strong satisfaction | Satisfaction levels with telephone and video communication were high across all facilities, but long-term care & nursing home participants were especially satisfied with video calls. | Increased social support | Not reported | Not reported |  |
|  |  |  |  | Increased connectedness |  |  |  |
| Scheerman, J.F.M. et al [[61](#_ENREF_61)] | Not reported | Not reported | Involving the mothers of adolescents into the intervention strengthened the outcomes. Intention, self-efficacy, perceived social support, self-monitoring, and coping planning influenced the intervention's effect on behavior change. Mobile applications particularly effective with younger generations given the time on average spent using applications. | Increased social support | Not reported | Not reported |  |
|  |  |  |  | Improved standard of care |  |  |  |
| Schrauben, S.J. et al [[62](#_ENREF_62)] | Not reported | Not reported | Younger participants, participants with a high school education or higher, and participants with higher household income were more likely to be current users of technology. Health literacy increased likelihood of technology use, especially internet, email, smartphone, and mHealth apps. Non-White participants and younger participants reported a higher level of interest in using digital and mHealth technology in the future. | Health literacy | Non-Hispanic Black participants reported less internet and email use than Non-Hispanic White participants. Older age, lower disease self-efficacy scores, and an annual income of less than $20,000 were associated with inadequate eHealth literacy. Concerns regarding the intrusiveness of the technology's monitoring features, a loss of in-person interaction, and uncertainty about future technologies were reported by participants, especially those over 65 years of age. Using technology was hindered by any physical limitations participants had and some reported requiring assistance or access in using technology. | Technical literacy |  |
|  |  |  |  | Education |  | Health literacy |  |
|  |  |  |  |  |  | Confidentiality / security |  |
| Shareef, M.A. et al [[63](#_ENREF_63)] | Not reported | Not reported | Trust is a key factor in determining whether or not elderly participants were willing to accept an autonomous homecare system. Personal ability and control impacts trust development in autonomous systems. Empathetic cooperation and social interaction contributes significantly to level of trust towards autonomous systems in the elderly. If elderly individuals believe they are able to use and control an autonomous homecare system and create some kind of empathetic connection, they are more likely to develop a sense of trust towards the system. | Enabled social interaction | Elderly autonomous homecare system users will perceive a risk in using new technologies. | Confidentiality / security |  |
|  |  |  |  | Increased social support |  | Technical literacy |  |
|  |  |  |  |  |  | Perceived lack of usefulness |  |
| van Dijk, M.R. et al [[64](#_ENREF_64)] | Not reported | Not reported | Use of the empowering intervention had a high level of compliance and increased intake of vegetables among participants. | Improved health behaviors | Not reported | Not reported |  |
|  |  |  |  | Increased adherence |  |  |  |
|  |  |  |  |  |  |  |  |
